# The anterior sylvian point as a reliable landmark for the anterior temporal lobectomy in mesial temporal lobe epilepsy: technical note, case series, and cadaveric dissection

**DOI:** 10.3389/fmed.2024.1352321

**Published:** 2024-06-20

**Authors:** Arianna Fava, Serena Vittoria Lisi, Luigi Mauro, Roberta Morace, Marco Ciavarro, Nicola Gorgoglione, Giandomenico Petrella, Pier Paolo Quarato, Giancarlo Di Gennaro, Paolo di Russo, Vincenzo Esposito

**Affiliations:** ^1^IRCCS Neuromed, Pozzilli, Italy; ^2^Laboratory of Neuroanatomy “G. Cantore”, IRCCS Neuromed, Pozzilli, Italy; ^3^Department of Human Neurosciences, University of Rome “La Sapienza”, Rome, Italy

**Keywords:** mesial temporal lobe epilepsy, anterior temporal lobectomy, anterior sylvian point, temporal anatomy, cadaveric dissection

## Abstract

**Introduction:**

Mesial temporal lobe epilepsy (MTLE) is one of the most prevalent forms of focal epilepsy in surgical series, particularly among adults. Over the decades, different surgical strategies have been developed to address drug-resistant epilepsy while safeguarding neurological and cognitive functions. Among these strategies, anterior temporal lobectomy (ATL), involving the removal of the temporal pole and mesial temporal structures, has emerged as a widely employed technique. Numerous modifications have been proposed to mitigate the risks associated with aphasia, cognitive issues, and visual field defects.

**Methods:**

Our approach is elucidated through intraoperative and cadaveric dissections, complemented by neuroradiological and cadaveric measurements of key anatomical landmarks. A retrospective analysis of patients with drug-resistant MTLE who were treated using our ATL technique at IRCCS Neuromed (Pozzilli) is presented.

**Results:**

A total of 385 patients were treated with our ATL subpial technique anatomically focused on the anterior Sylvian point (ASyP). The mean FU was 9.9 ± 5.4 years (range 1–24). In total, 84%of patients were free of seizures during the last follow-up, with no permanent neurological deficits. Transient defects were as follows: aphasia in 3% of patients, visual field defects in 2% of patients, hemiparesis in 2% of patients, and cognitive/memory impairments in 0.8% of patients. In cadaveric dissections, the ASyP was found at a mean distance from the temporal pole of 3.4 ± 0.2 cm (range 3–3.8) at the right side and 3.5 ± 0.2 cm (3.2–3.9) at the left side. In neuroimaging, the ASyP resulted anterior to the temporal horn tip in all cases at a mean distance of 3.2 ± 0.3 mm (range 2.7–3.6) at the right side and 3.5 ± 0.4 mm (range 2.8–3.8) at the left side.

**Discussion:**

To the best of our knowledge, this study first introduces the ASyP as a reliable and reproducible cortical landmark to perform the ATL to overcome the patients’ variabilities, the risk of Meyer’s loop injury, and the bias of intraoperative measurements. Our findings demonstrate that ASyP can be a safe cortical landmark that is useful in MTLE surgery because it is constantly present and is anterior to risky temporal regions such as temporal horn and language networks.

## Introduction

Mesial temporal lobe epilepsy (MTLE) is one of the most prevalent forms of focal epilepsy in surgical series, particularly among adults, and in 80% of the cases, the amygdalo-hippocampal complex is the seizure onset zone (1–6). Different techniques have been described to remove the mesial temporal epileptogenesis (7–14).

Selective approaches, such as the amygdalohippocampectomy, are targeted at mesial temporal structures preserving the lateral neocortex to decrease the risk of neurological issues, especially aphasia and cognitive deficits in the case of the dominant hemisphere (13, 15, 16). Nevertheless, several clinical research studies demonstrated that the temporal pole cortex (TPC) plays a crucial role in the temporal lobe seizure networks, in particular, in genesis and propagation (17–21). Moreover, some controversies about the selectivity and efficacy of different selective and non-selective surgical resections are still under debate (4, 14, 22, 23).

However, anterior temporal lobectomy (ATL) still remains the first surgical choice in many centers to treat drug-resistant MTLE (10, 12, 24). Over the decades, starting from the pioneering study by Wilder Penfield in 1936 (25), several techniques for ATL have been published to minimize surgical and neurological risks, with particular attention on language and memory functions and visual field deficits due to close relationship with language-related fasciculi and optic radiation. Consequently, those techniques focused on the posterior limits of the temporal cortex resection, establishing a maximum length from the temporal pole tip of 3–4.5 cm and 4.5–6 cm in the dominant and non-dominant hemispheres, respectively (8–12).

Nevertheless, the standardized measurements chosen *a priori* do not take into account the volumetric and morphological variability among patients that could be overcome by an anatomical-based technique.

In the present study, the authors aim to describe the surgical technique, i.e., the ATL, used to safely perform in patients with MTLE, introducing the Anterior Sylvian Point (ASyP) as a safe and reliable posterior landmark to start surgical resection.

In fact, the ASyP, corresponding to the arachnoid enlargement of the Sylvian fissure (SyF) inferior to the retracted pars triangularis of the inferior frontal gyrus, represents a constant anatomical point with precise relationships with surrounding brain structures (26–30). A step-by-step intraoperative description is presented and clarified by cadaveric dissections and radiological findings. The clinical results are illustrated along with a video case.

## Methods

The medical charts of consecutive people with drug-resistant MTLE who underwent surgical treatment with ATL were retrospectively reviewed. All surgeries were performed by the senior author (V.E.) at the IRCCS Neuromed, Pozzilli (IS), Italy. The present study was approved by the ethics committee of our institution.

All patients underwent standard preoperative evaluation at the epilepsy center of our institution, which included clinical assessment, continuous long-term video-EEG recording, neuropsychological assessment, and 3 T-MRI scan. Before surgery, each case was discussed in a case conference by a multidisciplinary team, including epileptologists, neurosurgeons, neuroradiologists, and neuropsychologists.

The microsurgical procedure was performed using a microscope (Leica, Zeiss, Pentero, or Kinevo).

The neuroradiological evaluation was carried out on 20 normal brain MRIs (volumetric T1 3D) to check the relationship between the ASyP and the tip of the temporal horn. The images were reconstructed and evaluated with a Radiant DICOM Viewer. The distance between the plane passing through the ASyP (perpendicular to the major axis of the Sylvian fissure and perpendicular to the cortical surface) and the tip of the temporal horn was measured by counting the slices (and multiplying for the thickness) from the two planes in the coronal T1 sequences.

A postoperative tractography was established to visualize the relationship between the surgical cavity and the closest fasciculi [uncinate fasciculus (UF), inferior fronto-occipital fasciculus (IFOF), and optic radiation]. The diffusion images were acquired on a GE SignaHDxt scanner using a diffusion sequence. A DTI diffusion scheme was used, and a total of 32 diffusion sampling directions were acquired. The b-value was 1,000. The in-plane resolution was 1 mm. The slice thickness was 3 mm. DSI studio was used as a tractography software tool to reconstruct the fasciculi in the postoperative MRI.^1^ In brief, the raw dMRI images were first converted to Neuroimaging Informatics Technology Initiative (NIfTI) format, imported into DSI Studio (Step t3: Fiber Tracking & Visualization), corrected for eddy current distortion and motion distortion, and rotated to align with the AC-PC line before reconstruction. The resulting images were run with GQI-based group probabilistic analysis with the default setting for each patient to generate and standardize GQI-based diffusion indices (31). The tensor metrics were calculated using DWI with a b-value lower than 1750 s/mm^2^. We reconstructed the tracts using the default tracking parameters (qa tracking index, min length 10, max length 200, and terminate if 100,000 seeds). The tracts were generated using the AutoTrack approach by preset regions of interest (ROIs) of a template HCP-1065 implemented in DSIstudio.

**Figure 1:**
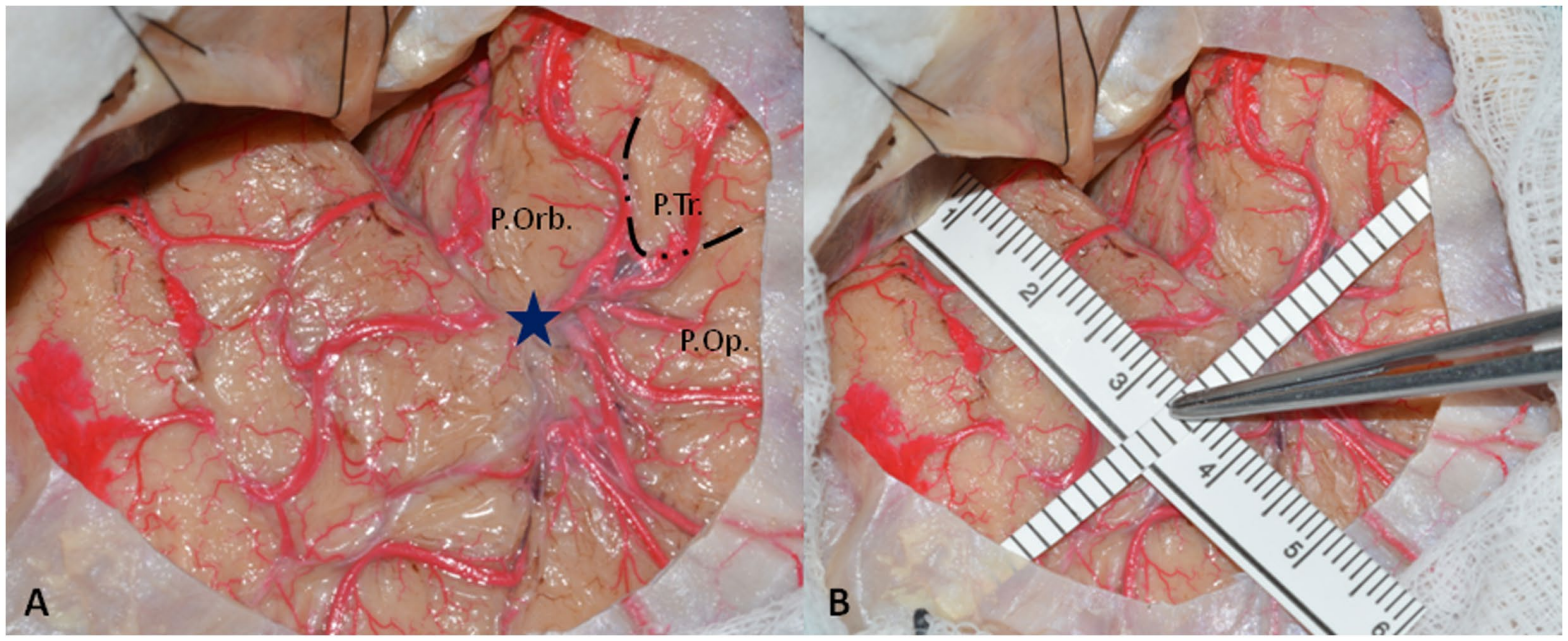
**(A)** Left-side minimal fronto-temporal craniotomy with exposure of pars orbitalis *(P.Orb.),* pars triangularis *(P.Tr.),* and pars opercularis *(P.Orb)* of inferior frontal gyrus and superior and middle temporal gyrus. The *dotted line* delineates the boundaries of pars triangularis that are constantly retracted without reaching the SyF. The *blue star* represents the ASyP localized on the arachnoidal enlargement below the pars triangularis. **(B)** The methods to perform the measurements from the temporal tip. One ruler is parallel to the SyF, touching the middle fossa anteriorly, and the other one is perpendicular to it, passing through the ASyP.

Cadaveric dissections were performed on 24 color-injected human embalmed specimens (48 sides) at the Laboratory of Neuroanatomy “G. Cantore,” Parco Tecnologico IRCCS Neuromed, Pozzilli (IS), Italy. For each specimen, after a minimal frontotemporal craniotomy centered on the squamosal part of the temporal bone, the distance between the tip of the temporal pole and ASyP was measured using a ruler, as shown in Figure 1. The ATL was performed entirely in two specimens (four sides).

White matter dissections have been performed to clarify the anatomy and the relationships between the ASyP and key anatomical structures encountered during the surgical procedure.

### Operative technique

**Figure 2:**
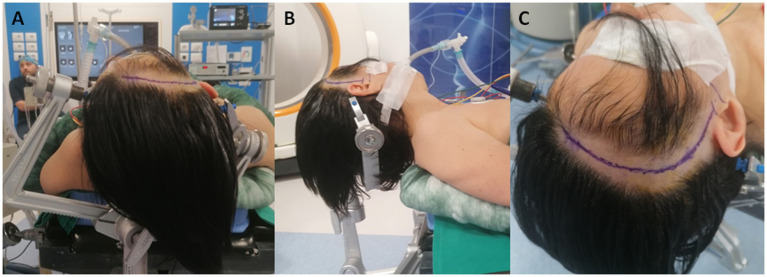
**(A,B)** Patient positioning with the head turned 30-degree contralaterally and extended. **(C)** Curvilinear fronto-temporal skin incision made behind the hairline and prolonged to the zygomatic root.

After general anesthesia has been induced, the patient is placed supine with the head fixed with a Mayfield head holder, turned 30-degree contralaterally, and extended (Figure 2). An antibiotic prophylaxis of 2 g of cefazolin is administered preoperatively.

A curvilinear fronto-temporal skin incision is made, and the musculocutaneous flap is retracted in one layer (Figure 2). After exposing the zygomatic root and the zygomatic process of the frontal bone, a minimal fronto-temporal craniotomy centered on the squamosal part of the temporal bone is performed.

**Figure 3:**
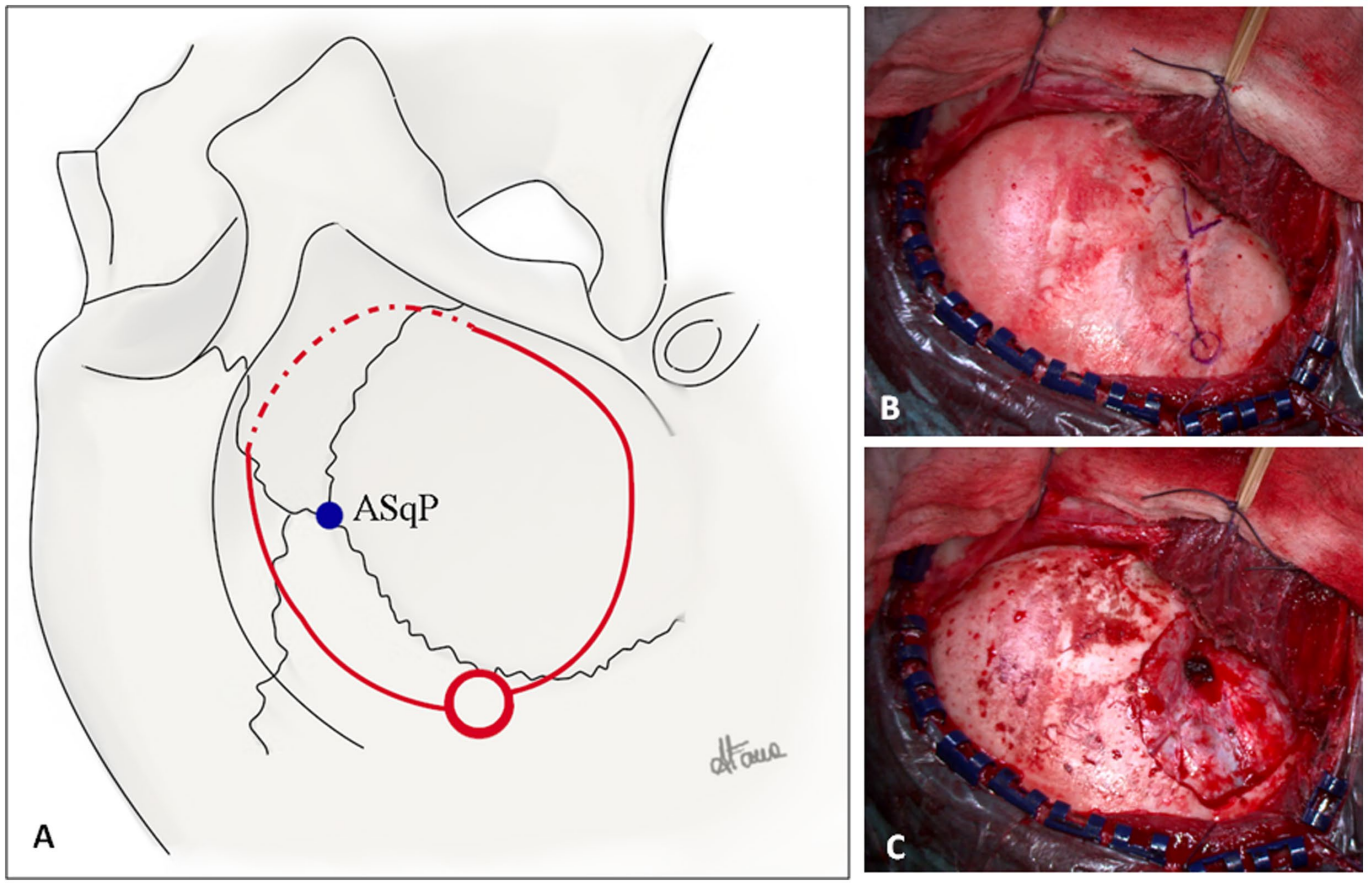
**(A)** Illustration of a right-side fronto-temporal craniotomy for ATL. The squamosal suture, pterion, coronal suture, and superior temporal line are illustrated. The *blue dot* represents the anterior squamosal point (ASqP) that corresponds to the anterior sylvian point (ASyP) on the cortical surface underneath the bone. The posterior burr hole is represented by the red circle. The superior and inferior cuts of the craniotomy *(continuous red lines)* are performed with the craniotome, whereas the anterior cut *(dotted red line)* is done using a 3-mm drill to keep bone as much as possible (Copyright Arianna Fava). **(B)** Intraoperative fronto-temporal exposure. The key anatomical landmarks are the zygomatic process of the frontal bone, the zygomatic root, pterion *(blue V),* ASqP, and the squamosal suture *(blue line).* **(C)** Dural exposure after minimal fronto-temporal craniotomy extended inferiorly to expose predominantly the anterior portion of the temporal lobe. The lesser wing is drilled and flattened.

A key craniometric point is the anterior squamosal point (ASqP), the most posterior part of the pterion, where the squamous suture intersects the sphenoparietal suture, underneath which the ASyP is located (27) (Figure 3A).

The burr hole is made approximately 2–3 cm posteriorly to the ASqP above the squamosal suture. In case of older patients, another burr hole can be placed above the zygomatic root. The frontal cut is made superiorly to the squamosal suture (bony landmark of the Sylvian fissure (SyF)) and inferiorly to the temporal line to expose the inferior frontal gyrus. The craniotomy must be as low and as anterior as possible to avoid unnecessary bone removal. Accordingly, the anterior cut is performed with a 3-mm course drill to thin the bone (Figures 3A,B). Thus, the craniotomy flap can be elevated and fractured. The residual lesser sphenoid wing is drilled and flattened to reduce the blind spot (Figure 3C). Further exposure is obtained by elevating the operating table to increase the anterior view.

**Figure 4:**
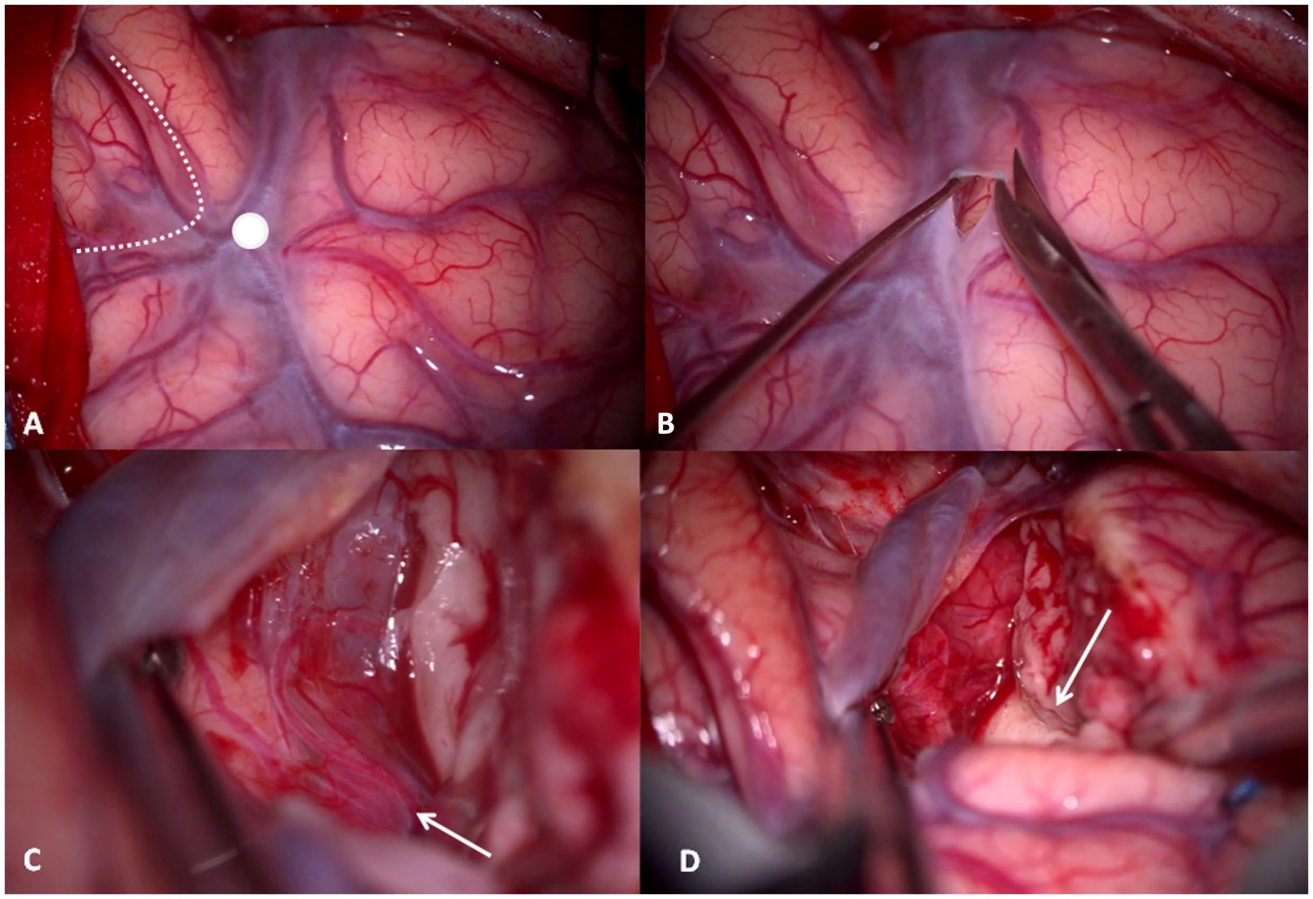
**(A)** Intraoperative right-side cortical exposure: SyF with the Sylvian veins, inferior to the superior temporal gyrus, superior to the inferior frontal gyrus. The *dotted line* delineates the boundaries of pars triangularis, and the *white dot* is the ASyP. **(B)** The arachnoid-pial layer is cut near to the SyF and anteriorly to ASyP. **(C)** Subpial technique: the temporal pole is separated from the pia mater, which protects the vessels within SyF and the frontal lobe. In transparency, the M1-M2 bifurcation *(white arrow)* corresponds to the limen insulae. **(D)** The mesial dissection of the temporal lobe from the uncus through the rhinal sulcus *(white arrow)*.

The dura is opened in a “C” shape and reflected anteriorly, showing the cortical anatomy with key landmarks: SyF, pars orbitalis, triangularis and opercolaris of the inferior frontal gyrus, and the ASyP. Inferiorly, superior and middle temporal gyri (T1 and T2, respectively) are exposed (Figure 4A).

Once the anatomy has been inspected, the arachnoid-pia layer is opened at the level of T1 anteriorly to the ASyP, 3 mm inferior, and parallel to the SyF (Figure 4B). The pia mater is grasped and elevated, and the brain is gently separated with a Penfield dissector and aspiration from the pia layer that covers the vessels of the Sylvian fissure and the frontal gyrus (subpial technique) (Figures 4C,D). Through the pia mater in transparency, the branches of the middle cerebral artery (MCA) can be identified, in particular, the M1-M2 bifurcation corresponding to the limen insulae (Figure 4C). This point is crucial because it represents our posterior limit in depth to avoid injury posteriorly and medially to the UF/IFOF complex and lenticulostriate arteries. The subpial dissection proceeds anteriorly and medially until reaching the rhinal sulcus, the anterior projection of the collateral sulcus that divides the temporal pole from the uncus (Figure 4D).

**Figure 5:**
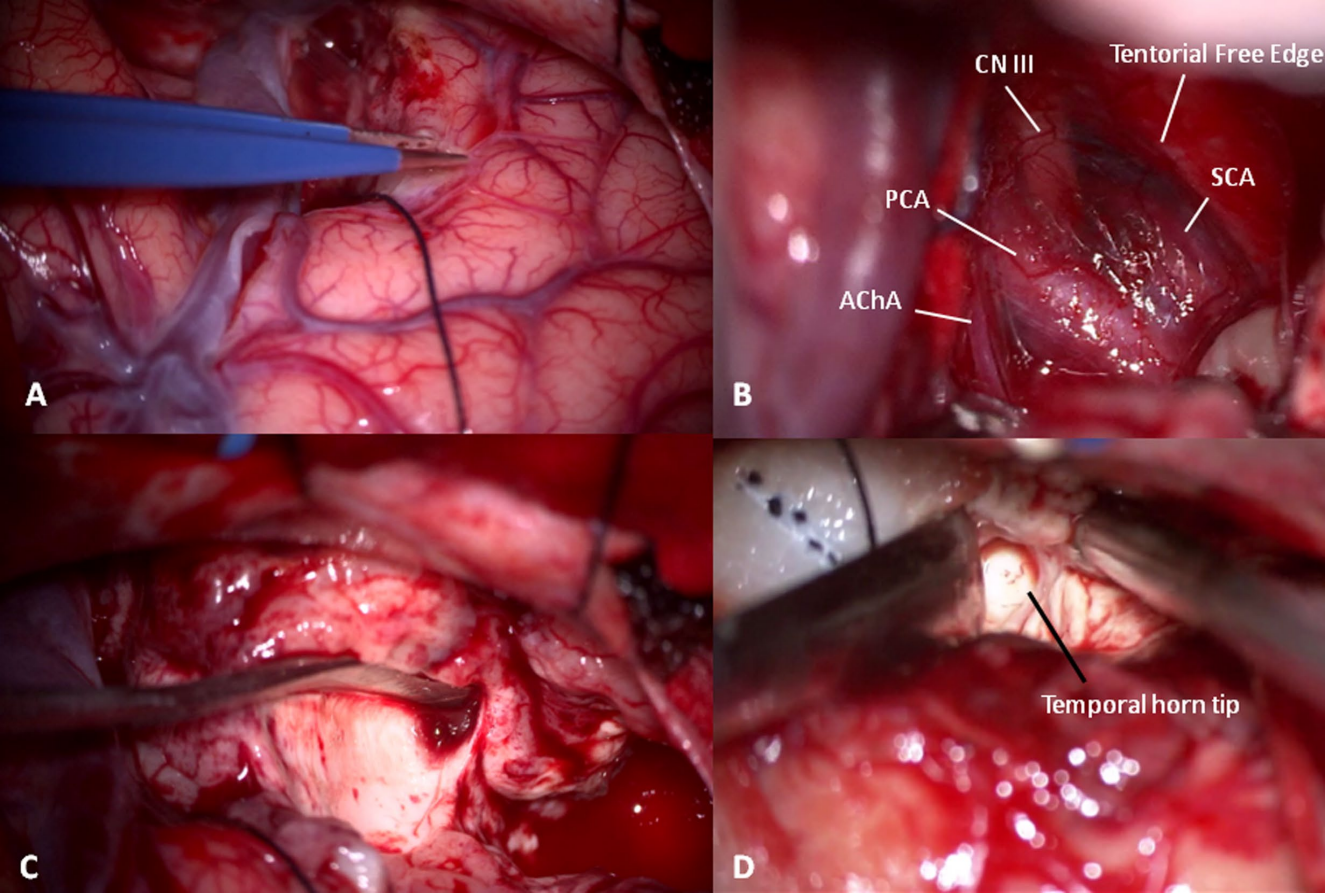
**(A)** Temporal pole corticectomy following a plane passing anteriorly to ASyP perpendicular to the temporal floor. **(B)** After temporal pole and uncus elevation in a subpial fashion, cisternal anatomy can be recognized: the third cranial nerve *(CN III)* under the free edge of tentorium between the superior cerebellar *(SCA)* and posterior cerebral arteries *(PCA)* and the choroidal artery (AChA). **(C)** White matter disconnection to remove the temporal pole. **(D)** Gentle dissection of temporal white matter with a Penfield dissector and aspirator from an anterior to posterior trajectory using the space left by the temporal pole removal to localize the temporal horn tip.

Then, the corticectomy continues from ASyP inferiorly following a plane perpendicular to the major axis of the Sylvian fissure and perpendicular to the cortical surface (Figure 5A). The temporal pole is disconnected from the pia mater at the base, exposing the free edge of the tentorium and freeing the pole that is resected, leaving the uncus below. The uncus is dissected from the pia mater and arachnoid, which protect the neurovascular structures medially that can be identified in transparency: internal carotid artery, posterior cerebral artery, anterior choroidal artery, lateral surface of the brainstem, superior cerebellar artery, and the third cranial nerve (Figure 5B). Finally, the pole is amputated and removed. Usually, the plane of resection described falls just anterior to the temporal horn tip. Starting from the amputated temporal white matter between T1 and T2 and following an antero-posterior trajectory, a careful dissection of the temporal white matter is performed with the aspirator and Penfield dissector to enter the anterior wall of the temporal horn of the lateral ventricle (Figure 5C).

Once identified, a cottonoid is put inside and reflected superiorly with the aim of protecting the optic radiation of Meyer’s loop situated at the roof and lateral wall of the temporal horn (Figure 5D).

**Figure 6:**
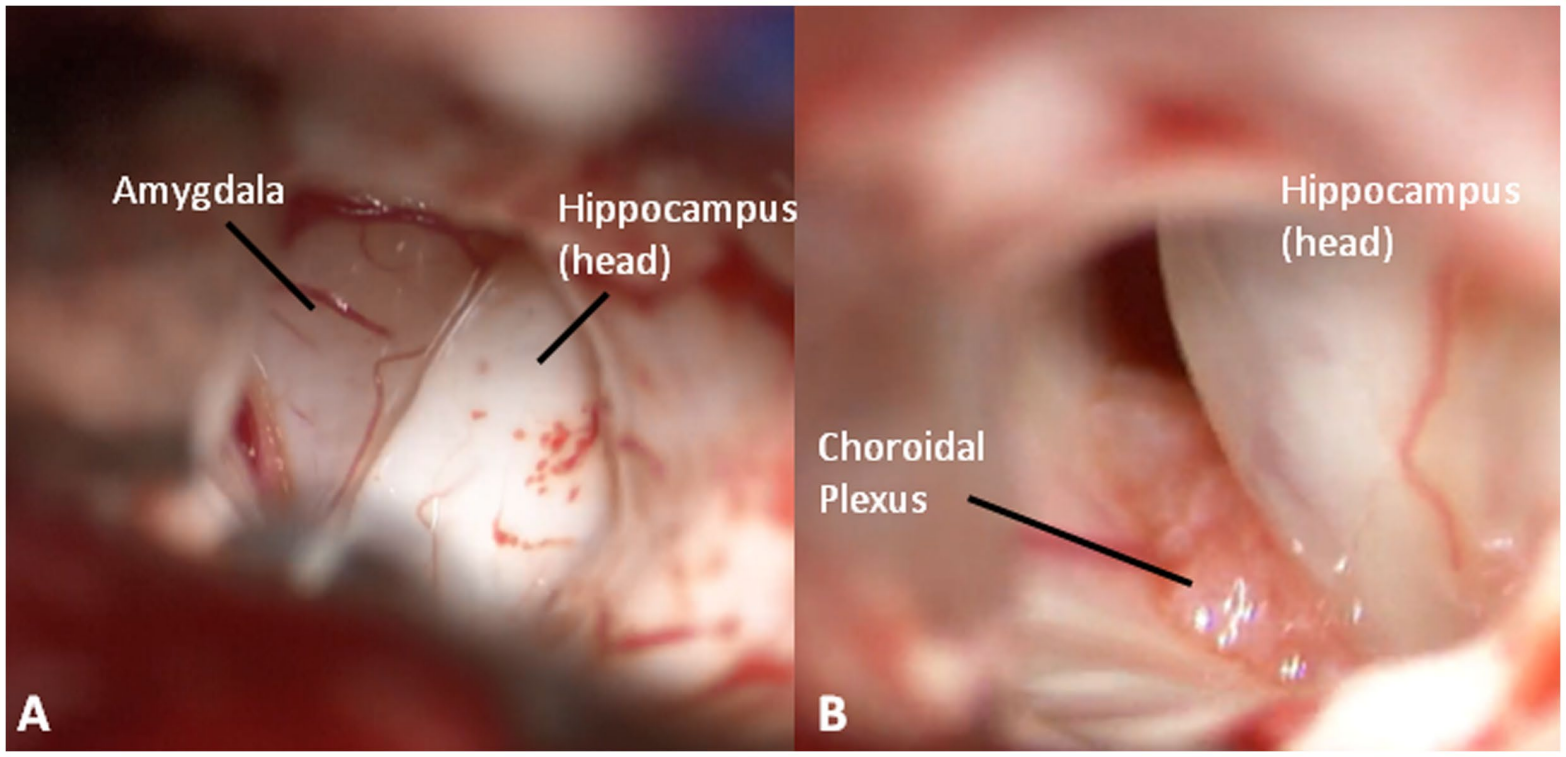
**(A)** An inside view of the anterior temporal horn, medially (greyish) the amygdala, and laterally (whitish) the hippocampus. **(B)** The choroidal plexus between amygdala and hippocampus.

**Figure 7:**
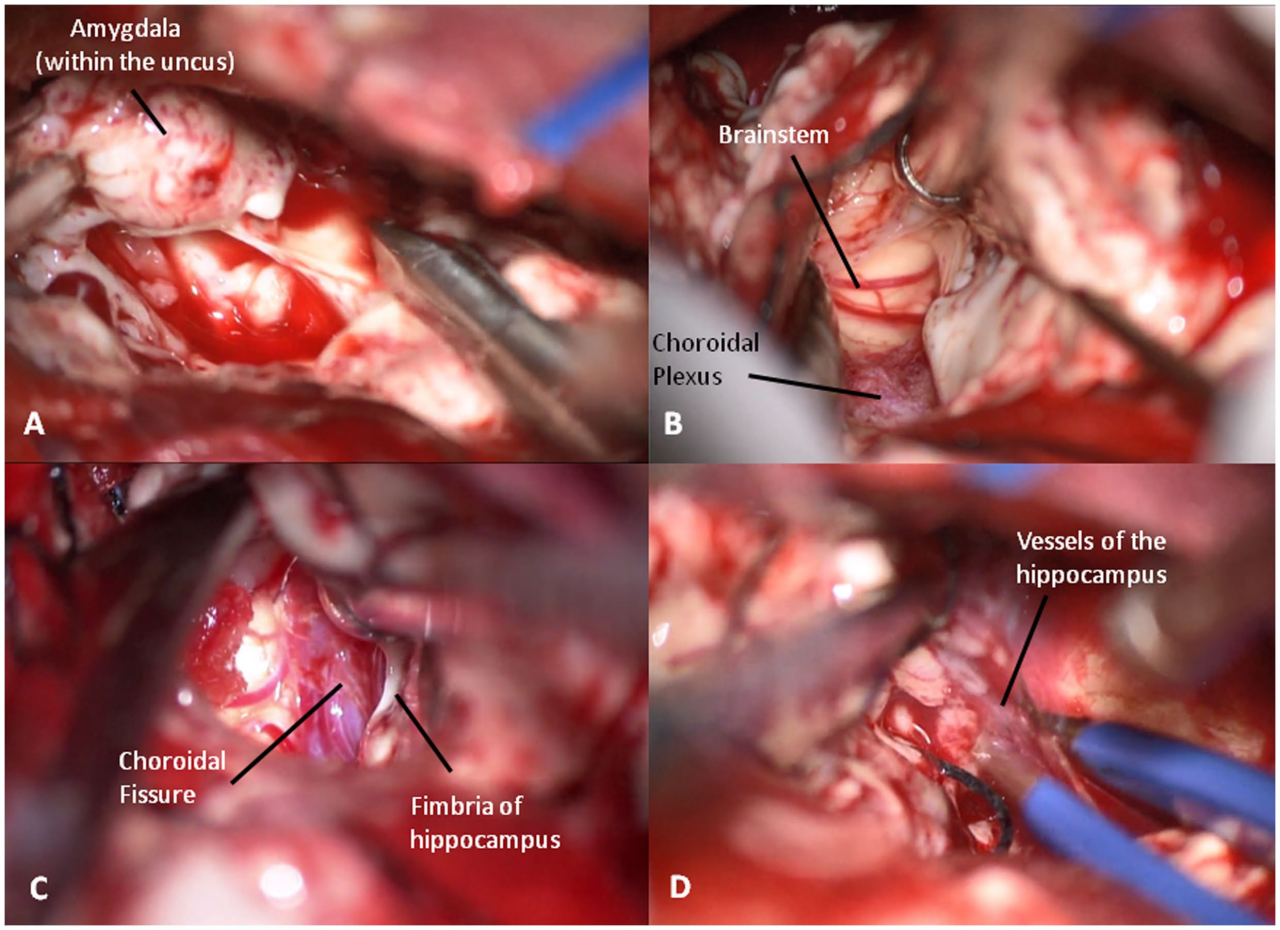
**(A)** The amygdale within the uncus is removed in a piecemeal fashion. **(B)** Brainstem decompression after the removal of the uncus. **(C)** Choroidal fissure is identified anterior to the choroidal plexus and medially to the hippocampus. **(D)** Coagulation of the vessels entering into the hippocampus to complete its resection.

At this point, looking medially inside the temporal horn, the two structures of the mesial temporal lobe, contained inside the uncus, can be identified: the grayish amygdala medially and the whitish head of hippocampus laterally, firmly stuck together (Figure 6). The amygdala is thus removed in a piecemeal fashion to decompress the medial part of the uncus (Figures 7A,B) until the optic tract and the anterior choroidal artery parallel to it come into view.

Then, the choroidal fissure is identified immediately anterior to the choroidal plexus and is opened, paying maximal attention to the underlying anterior choroidal artery (Figure 7C). The hippocampus is dissected medially from the crural arachnoid sheet and laterally from the collateral sulcus. Thus, the head and body of hippocampus are removed in a subpial fashion while the tail is pulled anteriorly grapping it with a hook. Medially, the hippocampal arteries from the posterolateral choroidal artery and posterior cerebral artery are identified with a pial sheet entering into the hippocampus. Thus, they are coagulated and cut, making the hippocampus free to be removed (Figure 7D). The removal of the hippocampus is fully completed when the lateral and postero-lateral brainstem are visible after the tail resection.

A surgical video is provided (Video 1).

## Results

### Clinical series

Between the years 1999 and 2022, 385 adults with drug-resistant MTLE underwent ATL, as described above, at our Epilepsy Surgery Center in Neuromed Institute I.R.C.C.S. of Pozzilli (IS). Preoperative clinical and radiological characteristics are detailed in Table 1. The male/female ratio was (203/182 = 1.12), with a mean age at the time of surgery was 36.9 years (range 18–65 years).

**Table 1:** Preoperative clinical and radiological characteristics.

Characteristics	*N* (%)
Patients	385
Male/female	203/182
Mean age at surgery (range)	36.9 years (18–65)
Epilepsy duration	
≤ 20 years	197 (51.2)
> 20 years	188 (48.8)
No. of AEDs prior to surgery	
1 AED	47 (12.2)
2–3 AEDs	307 (79.7)
≥ 4 AEDs	31 (8.1)
Dominant hand	
Right	351(91.2)
Left	26 (6.7)
Ambidextrous	8 (2.1)
Neurological status	
Normal	363 (94.3)
Motor and/or sensory impairments	5 (1.3)
Cognitive impairment	16 (4.1)
Motor and cognitive impairments	1 (0.3)
MR imaging findings	
Normal MR imaging	11 (2.9)
Hippocampal sclerosis	254 (66.0)
Focal cortical dysplasia	45 (11.7)
Atrofy and/or gliosis	12 (3.1)
Lesions (tumoral or vascular)	59 (15.3)
Others (e.g., traumatic, infectious, ischemic pathologies)	4 (1.0)
Side of anterior temporal lobectomy	
Right	187 (48.6)
Left	198 (51.4)

AED, antiepileptic drugs.*p-value statistically significant.

In our study population, patients used an average of two AEDs preoperatively.

In 51.2% of all included patients, the history of epilepsy was longer than 20 years. The majority of patients (91.2%) were right-handed, whereas 6.7% of patients were light-handed and 2.1% were ambidextrous. We performed right-sided ATL in 187 patients (48.6%) and left-sided ATL in 198 patients (51.4%). The mean postoperative follow-up was 9.9 ± 5.4 years (range 1–24). No neurological deficits occurred in 352 patients (91.4%). We observed speech disturbance in 12 patients (3.1%), visual field deficit in 9 patients (2.3%), hemiparesis in 8 patients (2.1%), and cognitive and/or memory disturbance in 3 patients (0.8%). In all cases, neurological deficits were transient, with a complete remission within 12 months postoperatively. Postoperative results are reported in Table 2. There was no significant correlation between postoperative neurological deficits and the side of surgery, except for speech disorders, which are encountered after left-side ATL only (*p* < 0.001) (Table 3). In our study, the mean follow-up was 9.9 years (range 1–24). Overall, at the time of the last follow-up, 322 patients (83.6%) were in Engel Class I (free of seizures), 33 patients (8.6%) were in Engel Class II (rare disabling seizures), 20 patients (5.2%) were in Engel Class III (worthwhile improvement), and 10 patients (2.6%) were in Engel Class IV (no improvement).

**Table 2:** Postoperative results.

Characteristics	*N* (%)
Mean FU (range)	9.9 ± 5.4 years (1–24)
Neurological status	
Normal	352 (91.4)
Motor impairment	8 (2.1)
Sensory impairment	1 (0.3)
Speech disturbance	12 (3.1)
Visual field deficits	9 (2.3)
Cognitive and/or memory impairments	3 (0.8)
Histological findings	
Normal findings	8 (2.1)
Hippocampal sclerosis	259 (67.3)
Focal cortical dysplasia	38 (9.9)
Atrofy and/or gliosis	20 (5.2)
Lesion (tumoral or vascular)	59 (15.3)
Others (e.g., traumatic, infectious, and ischemic pathologies)	1 (0.2)
Engel Class at last FU	
I (free of seizures)	322 (83.6)
II (rare disabling seizures)	33 (8.6)
III (worthwhile improvement)	20 (5.2)
IV (no improvement)	10 (2.6)

FU, Follow-up.

**Table 3:** Correlation between the ATL side and postoperative neurological complications.

	Right-side ATL (*N* = 187; 48.6%)	Left-side ATL (*N* = 198; 51.4%)	*p*-value
No postoperative neurological deficits	174 (93.0%)	178 (89.8%)	0.207
Postop neurological status			
Motor impairment	6 (3.2%)	2 (1.0%)	0.272
Sensory impairment	1 (0.5%)	0	0.303
Speech disturbance	0	12 (6.1%)	0.000*
Visual field deficit	5 (2.7%)	4 (2.0%)	0.671
Cognitive and/or memory impairments	1 (0.5%)	2 (1.0%)	0.596

ATL, Anterior Temporal Lobectomy.*p-value statistically significant.

### Radiological observations

In total, 40 brain hemispheres were evaluated and reconstructed with 3D MPR. In all hemispheres, the “M sign” corresponding to the inferior frontal gyrus (pars orbitalis, triangularis, and opercularis) was identified. On the right side, the tip of the temporal horn was identified at 3.16 ± 0.33 mm (range 2.7–3.6) posteriorly to the plane passing through the ASyP described above, while on the left side, it was identified at 3.46 ± 0.35 mm (range 2.8–3.8). In none of the cases, the temporal horn tip was found anteriorly to this plane.

**Figure 8:**
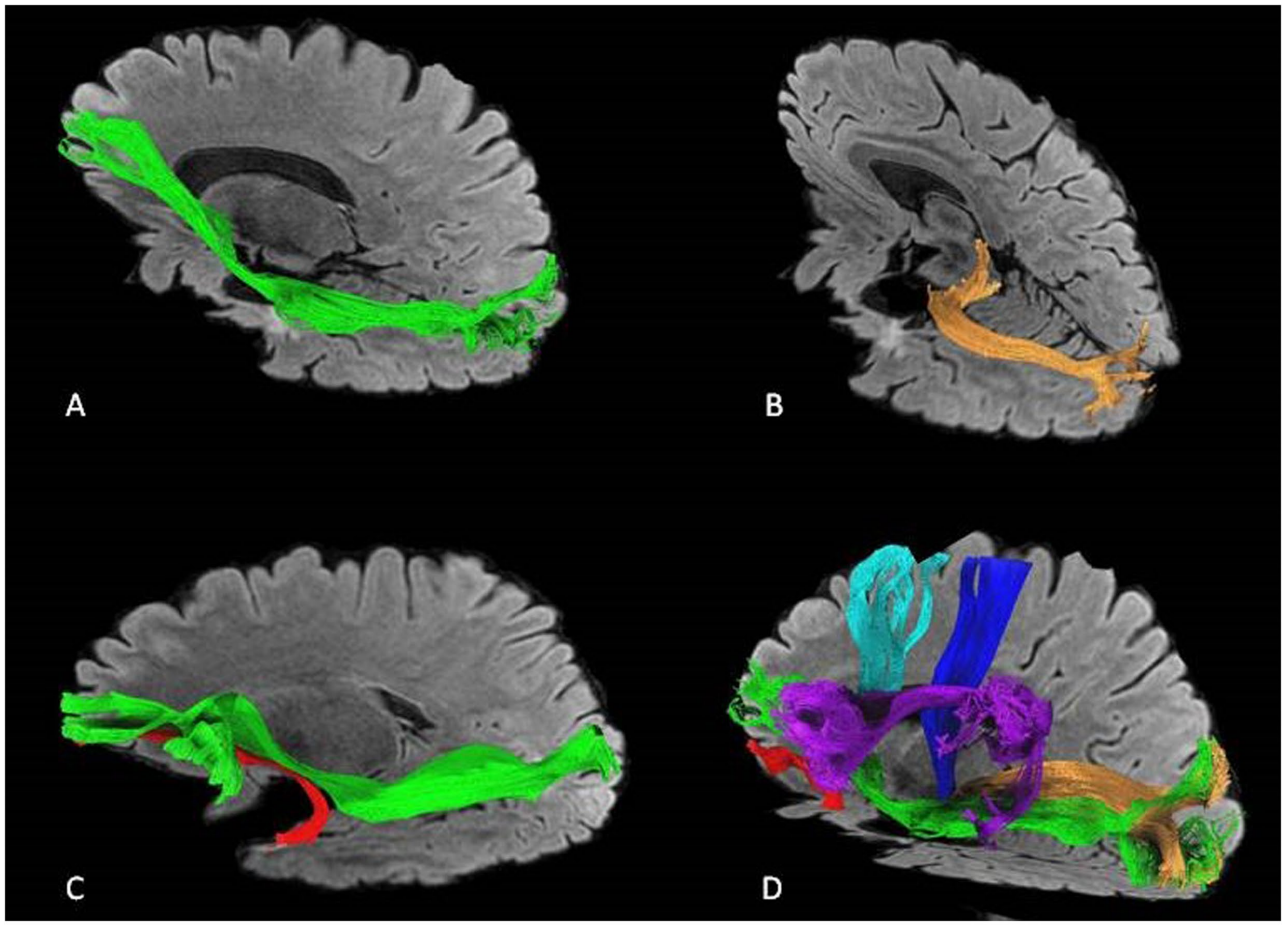
An illustrative case of postoperative tractography after left-side ATL showing the relationship between the surgical cavity and the main fascicule. **(A)** IFOF *in green,* **(B)** optic radiations *in orange,* **(C)** IFOF and UF *(uncinate) in red.* **(D)** IFOF, UF, optic radiations, AF *(arcuate) in purple*, corticospinal tract *in blue*, and fronto aslant tract *in light blue.*

An illustrative case of postoperative tractography with the main fasciculi closer to the surgical cavity is shown in Figure 8.

### Cadaveric dissection results

After performing the described craniotomy and opening the dura mater, the ASyP was identified in all the specimens for a total of 48 points. On the right side, the mean distance of ASyP from the temporal pole resulted in 3.4 ± 0.2 cm (range 3–3.8), and on the left side, it resulted in 3.5 ± 0.2 cm (3.2–3.9).

**Figure 9:**
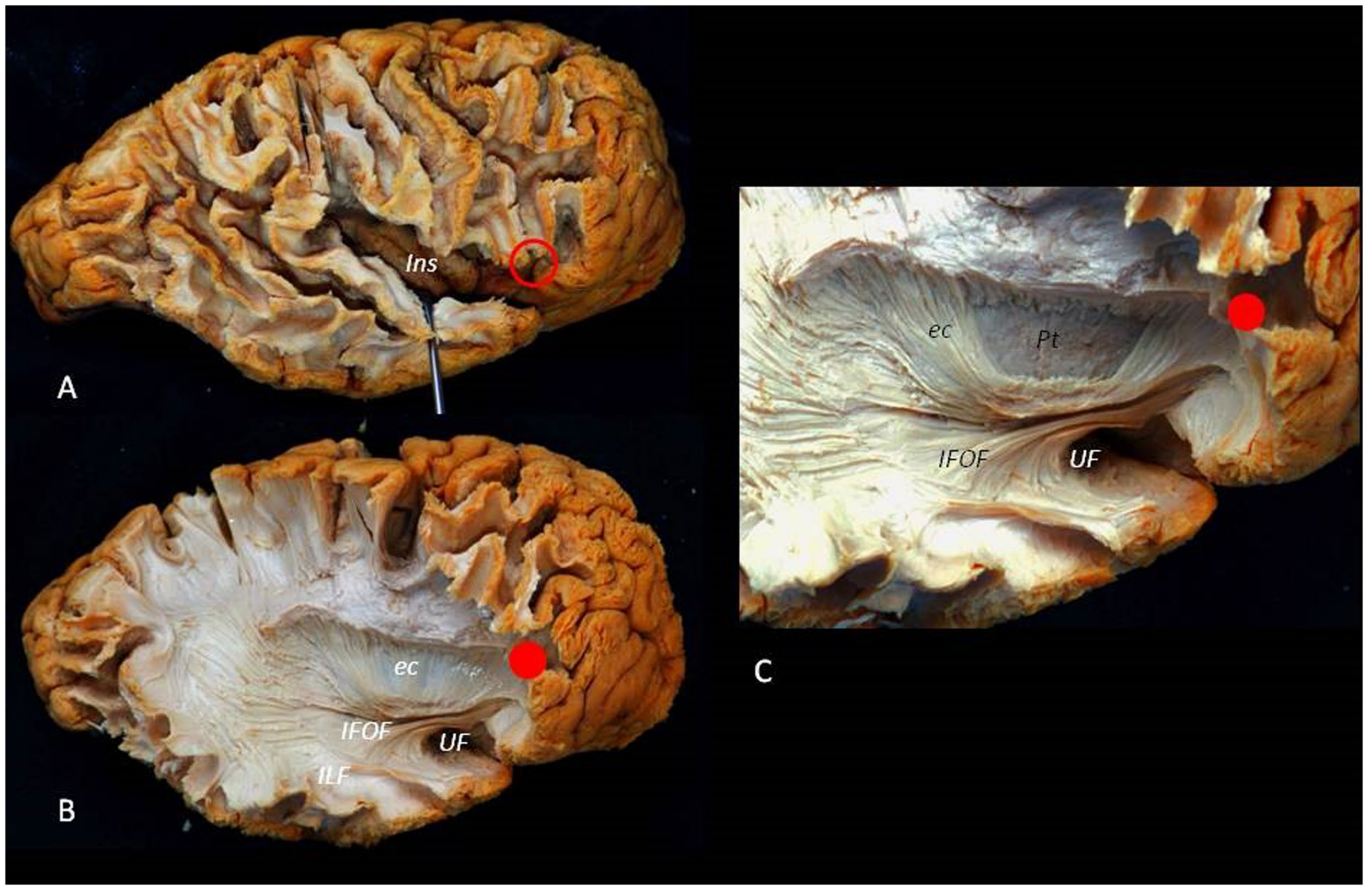
Lateral to medial dissection of a right hemisphere. **(A)** The cortex and U fibers of the frontal, temporal, and parietal lobes are partially removed. The *red circle* identifies the ASyP below the pars triangularis of the inferior frontal gyrus. By opening the SyF, the insula can be exposed, appreciating the relationship between the ASyP and the insular apex in depth. **(B)** The frontal, temporal, and parietal operculum have been removed. The IFOF and UF are dissected, revealing their relationship with the ASyP. ILF is also revealed. **(C)** Detail of IFOF and UF. Red dots indicate the relation between ASyP position in the cortical layer and subcortical structures. c, external capsule; Pt, Putamen; ILF, inferior longitudinal fasciculus.

Figure 9 illustrates the complex anatomy and the relationship between the ASyP and key anatomical structures encountered during the ATL.

## Discussion

MTLE is the most common form of drug-resistant focal epilepsy for which resective surgery is recommended as the treatment of choice (6, 32–36). Nevertheless, surgical option continues to be delayed and underused due to concerns related to invasiveness, surgical risks, and efficacy (37, 38). However, advances in diagnosis, neuroimages, and surgical techniques have greatly improved the safety, epilepsy outcome, and quality of life (32, 39).

Surgical options for drug-resistant MTLE can vary according to presurgical epilepsy evaluation, multidisciplinary discussion, and surgeons’ preference (2, 3, 9, 11, 17, 21, 34, 35, 40). The ATL, which consists of removing not only the mesial temporal lobe structures (amygdala, hippocampus, and parahippocampal gyrus) but also the temporal pole and anterior part of the lateral temporal neocortex, still remains to be one of the main surgical techniques despite other more selective approaches. Although controversies persist regarding epilepsy and neurological outcomes between ATL and selective approaches, it has been demonstrated that the temporal pole cortex plays a crucial role in temporal lobe seizure networks, particularly in genesis and propagation (17–21). This justifies its removal in MTLE surgery.

Some variations of the ATL technique have been described over the years to reduce postoperative neurological deficits: (1) visual field defects due to optic radiation of Meyer’s loop (lower division) that is located on the roof and the lateral wall of the temporal horn of lateral ventricle, thus entering from the lateral cortex, usually a superior quadrantanopia can occur or hemianopsia if the access is too posterior (due to the added injury of the upper division of optic radiation) (41–43); (2) semantic aphasia consequent to an injury of ventral language network (uncinate, middle and inferior longitudinal, and inferior fronto-occipital fasciculus) in the dominant hemisphere (44–48); (3) verbal or visuo-spatial memory related to dominant or non-dominant hippocampal total removal, respectively (49, 50).

In 1984, Spencer et al. (8) published their results using a modified ATL for MTLE consisting of minimizing surgical removal of the posterior temporal cortex. In particular, they defined the subpial temporal pole removal up to 4.5 cm from the temporal tip in the non-dominant hemisphere and 3 cm at the level of superior temporal gyrus in the dominant hemisphere, based on observations using stimulation in awake patients in which fluent speech arrest was never found anteriorly than 5.5 cm and 4.5 cm on the middle and temporal gyrus, respectively. Due to lateral temporal cortex sparing, Spencer et al. observed a significant reduction in visual field deficits, aphasia, and memory troubles (8).

Nowadays, Spencer’s technique is one of the most commonly used, although some variations among surgeons still exist in the following: (a) the maximum length from the tip of the temporal pole varying from 3 to 4.5 cm and 4.5 to 6 cm in dominant and non-dominant hemisphere, respectively; (b) the preservation or the partial removal of the superior temporal gyrus; (c) and the extent of hippocampal resection from anterior to posterior (8–12). Nevertheless, the current trend is minimizing temporal neocortical removal while resecting total mesial temporal structures (9, 10, 40).

To the best of our knowledge, this study is the first to introduce the ASyP as a reliable and reproducible cortical landmark to perform the ATL based on the anatomy to overcome the patients’ variabilities, the risk of Meyer’s loop injury, and the bias of intraoperative measurements. The ASyP represents a constant landmark in the cerebral surface identified as an arachnoidal enlargement of the Sylvian fissure located inferior to the retracted triangular part and antero-inferior to the opercular part of the inferior frontal gyrus (26, 27, 29, 51, 52). It divides the Sylvian fissure into its main anterior and posterior rami, and it is commonly used as a starting point to open the Sylvian fissure (29, 51). However, as reported by Ribas et al. (27), the striking cisternal appearance of the ASyP can be used intraoperatively to identify also important neural and sulcal structures usually hidden by arachnoidal and vascular coverings. In fact, opening the Sylvian fissure at the level of the ASyP conducts directly to the insular apex, identifying the most posterior branch of M2 (28, 30). The limen insulae with the MI-MII bifurcation are located a little deeper and anterior, 10–20 mm perpendicular to the ASyP itself (27, 29). As described above, the limen insulae are crucial because they represent our posterior limit in depth to avoid injury posteriorly and medially to the UF/IFOF complex and lenticulostriate arteries (53).

In both cadaveric specimens (48 sides) and all patients of our series, the ASyP was consistently identified as a clear landmark. In cadaveric specimens, the mean distance from the temporal pole to the ASyP was 3.4 cm (range 3–3.8) on the right side and 3.5 cm (3.2–3.9) on the left side, resulting within the maximum posterior distance allowed. Additionally, our radiological analysis of the relationship between the ASyP and the temporal horn tip showed that the temporal horn consistently lay posterior to the ASyP. This distance was 3.2 mm (range 2.7–3.6) on the right side and 3.5 mm (range 2.8–3.8) on the left side, ensuring a safe distance from Meyer’s loop.

These findings challenge the conventional posterior limit of ATL (4.5–6 cm, as described in the literature), suggesting that adhering to this range may risk accessing the temporal horn too laterally, increasing the risk of visual field deficits. Instead, our results support an approach entering the temporal horn through the temporal pole corridor with an anterior–posterior trajectory, allowing the protection of Meyer’s loop laterally and superiorly while working medially. In summary, the ASyP emerges as a reliable and consistently present cortical landmark, offering valuable guidance in MTLE surgery by being anterior to critical temporal regions such as the temporal horn and language networks.

Moreover, previous anatomical studies demonstrated that the localization of ASyP in the skull surface can be easily and properly predicted by craniometric points without using neuronavigation. In fact, the ASyP is located underneath the ASqP, which is the most posterior part of the pterion where the squamous suture intersects the sphenoparietal suture (27, 30). Thus, we can tailor a minimal fronto-temporal craniotomy focused on the squamous part of the temporal bone and on the anterior squamous point to reduce approach-related risks (venous infarction and brain manipulation) and cosmetic issues.

In our large series of adult patients who had undergone ATL using the described technique, we obtained a sustained long-term benefit, with most of the patients free of seizures, in line with the literature (34, 54, 55), and a low rate of neurological deficits, transient in all cases. In particular, we observed speech disturbance in 3.1% of patients (12 patients), visual field defects in 2.3% of patients (9 patients), hemiparesis in 2.1% of patients (8), and cognitive and/or memory disturbance in 0.8% of patients (3), slightly inferior to that reported in the literature (language disorder in 4% of patients, visual field defects in 6% of patients, hemiparesis in 4% of patients, and cognitive and memory disturbances in 5 and 7% of patients respectively) (56). Considering the risk of hemiparesis and language disorder, the subpial technique can help reduce the risk of vascular injuries on MCA branches and anterior choroidal artery, avoiding the opening of SyF (7, 29, 51). The pia layer is on the left side of the vessels to protect them not only from direct manipulation but also from surgical bleeding that can increase vasospasm and consequent cerebral ischemia.

In our series, patients who presented postoperative language disorders were all treated on the left side (*p* < 0.001), but none with a permanent deficit. Considering the distance from the temporal pole to the ASyP of approximately 3.5 cm and the non-negligible risk of language disturbance, attention has to be paid when resection is classically extended until 4.5 cm posterior to the temporal pole. Consequently, resting-state functional MRI (rs-fMRI) may be a potential perspective to add additional information during the preoperative surgical planning in the dominant hemisphere and to evaluate altered brain network architecture due to epileptogenic activity. This technique could also be useful for detecting postoperative brain network reorganization due to resective surgery.

Finally, a matter of discussion is the generalizability of this type of resection at all non-lesional temporal lobe epilepsy (TLE). Our approach in these cases is to proceed with ATL when the history of the disease is relatively short, and the electroclinical correlations indicate an early and limited involvement of the temporo-mesial structures (e.g., the presence at the onset of vegetative-affective aura, Ictal EEG pattern >5 Hz, PET indicating hypometabolism limited to temporal mesial structures). In all other cases, we proceed with intracranial investigation, and the resection is tailored to EEG findings. Therefore, we suggest that this approach cannot be applied systematically to all non-lesional epilepsy.

### Limitations

Although different methodologies and various investigative perspectives supported by clinical outcomes have been used to clarify the relationships among anatomical structures (ASyP, temporal horn, temporal pole, and fascicles), some limitations can affect the present study. As a retrospective study, reporting and recall bias can impact clinical outcomes and complication rates, which could be underestimated. Moreover, anatomical measurements on cadaveric specimens could be affected by approximations due to the absence of CSF and fixed parenchyma.

## Conclusion

MTLE is one of the most common causes of drug-resistant seizures for which surgery is recommended as the treatment of choice. Our technique, supported by radiological and cadaveric findings and our clinical experience, introduces the ASyP as a reliable, constant, and safe anatomical landmark to perform the ATL to overcome the patients’ variabilities, the bias of intraoperative measurements, and the risk of neurological deficits.

## Data availability statement

The raw data supporting the conclusions of this article will be made available under reasonable request to the corresponding author.

## Ethics statement

The studies involving humans were approved by Comitato Etico Istituto Neurologico Mediterraneo Neuromed – IRCCS Neuromed, Pozzilli. The studies were conducted in accordance with the local legislation and institutional requirements. Written informed consent for participation was not required from the participants or the participants’ legal guardians/next of kin in accordance with the national legislation and institutional requirements.

## Author contributions

AF: Writing – original draft, Methodology, Investigation, Formal analysis, Data curation, Conceptualization. SL: Writing – review & editing, Visualization, Validation, Investigation, Data curation. LM: Writing – review & editing, Visualization, Validation, Investigation, Data curation. RM: Writing – review & editing, Visualization, Validation, Supervision. MC: Writing – review & editing, Visualization, Validation, Software, Investigation, Data curation. NG: Writing – review & editing, Visualization, Validation, Supervision. GP: Writing – review & editing, Visualization, Validation. PQ: Writing – review & editing, Visualization, Validation, Supervision. GG: Writing – review & editing, Visualization, Validation, Supervision. PR: Writing – review & editing, Visualization, Validation, Supervision. VE: Writing – review & editing, Visualization, Validation, Supervision, Investigation, Formal analysis, Conceptualization.

## Footnotes

^1^ http://dsi-studio.labsolver.org
